# Better, not just fewer: Rethinking antibiotic prescribing

**DOI:** 10.1371/journal.pmed.1004941

**Published:** 2026-02-27

**Authors:** Giorgia Sulis, Derek R. MacFadden

**Affiliations:** 1 School of Epidemiology and Public Health, Faculty of Medicine, University of Ottawa, Ottawa, Ontario, Canada; 2 Methodological and Implementation Research Program, Ottawa Hospital Research Institute, Ottawa, Ontario, Canada; 3 The Ottawa Hospital, Ottawa, Ontario, Canada

## Abstract

Clinical decision support tools can curb unnecessary antibiotic use, but success depends on more than technology. In this Perspective, Giorgia Sulis and Derek MacFadden explore behavioral, equity, and governance challenges in tackling antimicrobial resistance, and why “better, not just fewer” prescriptions are essential.

## Teaser

Clinical decision support tools can curb unnecessary antibiotic use, but success depends on more than technology. Here, we explore behavioral, equity, and governance challenges in tackling antimicrobial resistance, and why “better, not just fewer” prescriptions are essential.

Antimicrobial resistance (AMR) is among today’s most pressing global health threats, responsible for millions of deaths annually and placing an enormous burden on health systems worldwide [1,2]. A major driver of this crisis is the inappropriate and often unnecessary use of antibiotics, particularly in primary care settings where most prescriptions originate. In these contexts, clinicians often face diagnostic uncertainty, limited access to laboratory testing, and overwhelming patient loads—factors that encourage precautionary prescribing even when antibiotics are not clinically indicated [3]. The challenge is especially acute in sub-Saharan Africa, where systemic resource constraints intersect with high infectious disease burdens [1]. Here, the consequences of unnecessary antibiotic use extend beyond individual patient harm to the population itself, where AMR is selected for and propagated, undermining treatment efficacy for common infections [3,4].

Among the strategies proposed to address this issue, clinical decision support tools have gained attention for their potential to improve prescribing practices. While promising, these tools are not a panacea; their success depends on integration within broader antimicrobial stewardship frameworks that combine technology with behavioral, organizational, and policy interventions [5,6].

In a recent *PLOS Medicine* study [7], Kulinkina and colleagues conducted a pragmatic, cluster non-randomized trial across 32 health centers in Rwanda, involving nearly 60,000 consultations with acutely ill children aged 14 years or younger [7]. The trial aimed to evaluate the impact of ePOCT+ (a digital clinical decision support algorithm that provides guidance on antibiotic prescribing practices in routine primary care) not only on whether to prescribe antibiotics, but also on drug selection (including first- and second-line options), dosing, and duration. The intervention was implemented through a tablet-based application and designed to assist clinicians (primarily nurses, who provide most pediatric outpatient care in Rwandan public health centers) in managing acute illness. The findings were striking: Antibiotic prescription rates fell from approximately 70% under standard care to about 25% with ePOCT+, without evidence for relevant negative clinical impacts [7]. While clinical failure and severe outcomes were slightly more frequent in some analyses, these differences were within the predefined non-inferiority margin and likely reflect residual imbalances between clusters—a fundamental limitation of the non-randomized design, which introduces potential for selection bias and confounding. Importantly, a parallel cluster randomized controlled trial of the same ePOCT+ intervention conducted in Tanzania demonstrated a nearly identical reduction in antibiotic prescribing without increased clinical failure [8], strengthening confidence in the causal effects observed in Rwanda.

Besides the large sample size, the incorporation of point-of-care tests like hemoglobin, C-reactive protein, and pulse oximetry alongside the inclusion of ongoing clinical mentorship to support uptake of the intervention were major strengths of the trial. However, important limitations remain: Incomplete intervention uptake (averaging around 70%, reflecting challenges in workflow integration and high staff turnover); reliance on clinician-reported prescribing data, which may have led to overestimated adherence and introduced potential reporting bias; and absence of adjustment for prescriber characteristics. Also, the study was not powered to detect rare adverse outcomes or unintended consequences. Despite these caveats, the trial provides compelling evidence of the potential for structured decision support to improve antibiotic stewardship in resource-limited settings.

While digital and guideline-based interventions hold promise for improving antibiotic stewardship, their success is far from guaranteed (see **Box 1**). Evidence from multiple settings shows that these tools often fail when implemented in isolation, largely because prescribing behavior is shaped by complex cognitive and systemic factors [5,6,9]. Clinicians operate under time pressure, diagnostic uncertainty, and patient expectations, all conditions that foster decision fatigue and reliance on trial and error, biased heuristics, or educated guesses [10,11]. Without proper training and complementary behavioral change strategies, even well-designed algorithms risk being sidelined.

**Box 1 Policy and practice implications**

Scaling up clinical decision support tools for antimicrobial stewardship requires more than technical deployment. Several policy and governance issues must be addressed to ensure safe, equitable, and sustainable implementation.

- **Governance and accountability:** Clear ownership of algorithms is essential. Who is responsible for maintaining, updating, and validating these tools? Without defined accountability, there is a risk of outdated recommendations, algorithmic bias, and misuse. Transparent governance structures should include health authorities, professional bodies, and independent oversight mechanisms.
- **Data ethics and privacy:** Digital tools rely on patient-level data, raising concerns about confidentiality and security, particularly in low-resource settings where regulatory safeguards may be weak. Policies must mandate secure data storage, consent protocols, and compliance with international standards for health data protection.
- **Financing models:** Many interventions begin as donor-funded pilots, but sustainability requires long-term domestic investment. Governments should explore blended financing models that combine public funding with strategic partnerships, while avoiding dependency on short-term external support.
- **Regulatory frameworks:** Robust regulation is critical to ensure quality, safety, and interoperability. National digital health strategies should incorporate standards for algorithm validation, integration with electronic medical records, and alignment with antimicrobial stewardship guidelines. Regulatory frameworks must also anticipate emerging technologies, such as artificial intelligence-driven decision support, to prevent gaps in oversight.

A key tension lies between algorithmic recommendations and clinician autonomy. Prescribers may override guidance when it conflicts with their clinical intuition, perceived patient needs, or past experiences. As such, interventions that ignore intrinsic motivation, professional identity, and trust dynamics in the provider–patient relationship are unlikely to achieve sustained impact. Designing systems that support, rather than constrain, clinical judgment is therefore essential.

Contextual adaptation is another critical determinant of success [12]. “One-size-fits-all” algorithms rarely perform optimally across diverse health systems [5]. Variations in disease epidemiology, population composition, resource availability, and workforce capacity demand locally tailored solutions. For example, it is intuitive that an algorithm calibrated for high malaria prevalence may misguide prescribing in regions where malaria is rare.

Furthermore, equity considerations must not be overlooked. Digital stewardship tools risk widening disparities if access is limited to well-resourced facilities or urban centers. Rural clinics, private-sector providers, and community pharmacies often remain outside the reach of such innovations. Gender dynamics also matter: Women, who constitute a large proportion of frontline health workers (especially nurses) in many low-income settings, may face unique barriers to training and technology adoption [13]. These barriers, combined with systemic resource gaps, could influence both uptake and effectiveness, introducing potential bias in real-world implementation. Ensuring equitable access and usability is therefore a prerequisite for meaningful scale-up.

Aiming for an overall reduction in antibiotic use is indeed a key goal, especially given the widespread use without clinical indication. Yet, antimicrobial stewardship is not only about reducing the overall volume of antibiotic prescriptions; it also requires ensuring that the right drug, dose, and duration are chosen for each clinical scenario. Efforts to reduce unnecessary prescribing should not overlook the importance of improving adequacy of those antibiotics that are ultimately given—with the aim of reducing treatment failure and improving infection outcomes amongst patients appropriately started on antibiotic therapy. Future trials should therefore complement overall prescription rates with measures of antibiotic appropriateness, such as adherence to guidelines for antibiotic choice and duration, or metrics aligned with WHO Access-Watch-Reserve (AWaRe) framework [14], a critical benchmark for rational antibiotic use. Composite indicators that jointly capture antibiotic spectrum and duration could further strengthen stewardship evaluations.

The trial by Kulinkina and colleagues [7] offers a unique opportunity to explore this dimension. In addition to measuring prescription rates, the study collected detailed data on antibiotic types and dosages, which could be analyzed to assess adherence to national guidelines and the AWaRe framework principles. Such analyses would provide valuable insights into whether reductions in prescribing were accompanied by improvements in appropriateness. However, early signals suggest persistent challenges. While the option for clinicians to reject algorithm recommendations or manually add diagnoses and medications was an intentional design feature of ePOCT+ aimed at preserving clinician autonomy, the study reported frequent manual overrides of algorithm recommendations, with approximately one-quarter of antibiotics prescribed not being those suggested by ePOCT+ [7]. Notably, this proportion represents 25% of an already substantially reduced overall antibiotic prescribing rate achieved by the intervention, rather than widespread divergence from algorithm guidance.

This pattern underscores the complexity of changing entrenched prescribing behaviors and highlights the need for complementary strategies. Training and supervision remain essential to reinforce guideline-based choices, while the refinement of algorithms may help address gaps in usability and trust among clinicians. Ultimately, stewardship interventions must go beyond curbing unnecessary prescriptions to promote rational selection and dosing. Without this dual focus, efforts to combat AMR risk falling short of their intended impact.

The next frontier in antimicrobial stewardship lies in integrating clinical decision support tools with broader health system innovations. Linking these tools to AMR surveillance platforms and infectious disease prevalence data could enable dynamic updates to prescribing guidance based on local epidemiology, informing both whether antibiotics are needed and which pathogens are most likely. Advances in predictive analytics and artificial intelligence offer opportunities for personalized antibiotic recommendations, but their deployment must be accompanied by rigorous evaluation and governance safeguards. Human factors research remains critical to understand clinician trust, patient expectations, and cultural norms that shape prescribing behavior. Expanding stewardship efforts beyond public facilities to include the broad and highly heterogenous range of private-sector providers, a major source of antibiotics in sub-Saharan Africa, is also crucial. Finally, adopting a One Health perspective can help translate lessons from human health into veterinary and agricultural contexts, addressing AMR across interconnected ecosystems.

Clinical decision support algorithms such as ePOCT+ show promise for improving prescribing practices and mitigating AMR. However, their success hinges on more than technology alone: It requires system-level integration, continuous monitoring of adherence, and sustained investment to ensure these tools become a cornerstone of comprehensive antimicrobial stewardship strategies.

## Abbreviations

AMR: antimicrobial resistance
AWaRe: Access-Watch-Reserve
